# Supplementary material for: Identification of photoperiod-induced specific miRNAs in the adrenal glands of Sunite sheep (Ovis aries)
Source: Front Vet Sci. 2022 Jul 22;9:888207. doi: 10.3389/fvets.2022.888207 (PMC9354845; doi:10.3389/fvets.2022.888207)
Supplement: Supplementary file 1 [file Data_Sheet_1.ZIP › Supplementary file/miRDeep2 file.zip!/miRDeep2 file/Know_Structure/LP42A2/oar-let-7c.pdf]

miRBase precursor : oar-let-7c
Total read count : 113445
oar-let-7c read count : 113398
remaining reads : 47

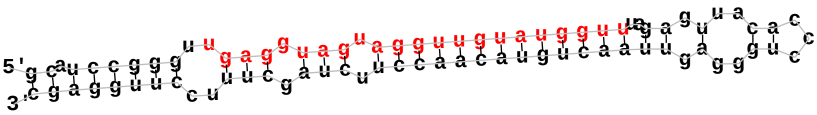

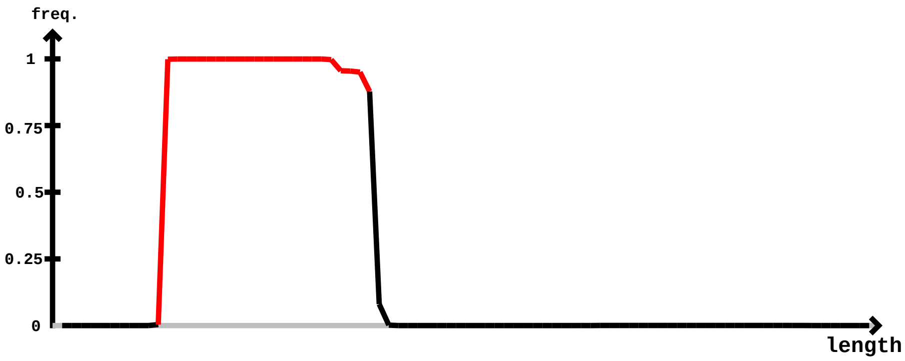

oar-let-7c

```
5'- gcauccgggu ugagguaguagguuguaugguu uagaguuacacccugggaguuaacuguacaaccuucuagcuuuccuuggagc   -3'    exp
    ((.((((((..(((.(((.(((((((((((((..((.(..((...))..).))))))))))))))).))).)))..))))))))   reads    mm    sample
    ........guugagguaguagguuguauggu.....................................................   3        0     seq
    ........guugagguaguagguuguaugguu....................................................   1        0     seq
    .........uugagguaguagguuguau........................................................   3        0     seq
    .........uugagguaguagguuguauA.......................................................   1        1     seq
    .........uugagguaguagguuguaug.......................................................   1        0     seq
    .........uugagguaguagguuguaugg......................................................   9        0     seq
    .........uugagguaguagguuguauAgu.....................................................   6        1     seq
    .........uugagguaguagguuguauggu.....................................................   143      0     seq
    .........uugagguaguagguuguaugguu....................................................   252      0     seq
    .........uugagguaguagguuguauAguu....................................................   10       1     seq
    .........uugagguaguagguuguaugguuu...................................................   15       0     seq
    .........uugagguaguagguuguauAguuu...................................................   1        1     seq
    ..........uUagguaguagguugua.........................................................   1        1     seq
    ..........ugaggCaguagguugua.........................................................   1        1     seq
    ..........ugagguaguagguugua.........................................................   239      0     seq
    ..........ugagguUguagguugua.........................................................   1        1     seq
    ..........ugagguaguaAguugua.........................................................   6        1     seq
    ..........ugagguagGagguugua.........................................................   28       1     seq
    ..........ugaAguaguagguugua.........................................................   1        1     seq
    ..........ugagguaguUgguugua.........................................................   1        1     seq
    ..........ugagguaguagguuguau........................................................   4249     0     seq
    ..........ugaAguaguagguuguau........................................................   31       1     seq
    ..........ugagguaguaAguuguau........................................................   18       1     seq
    ..........ugagguagGagguuguau........................................................   410      1     seq
    ..........ugagguUguagguuguau........................................................   1        1     seq
    ..........ugagguaguagguuUuau........................................................   7        1     seq
    ..........ugagguaguagguuguaug.......................................................   138      0     seq
    ..........ugagguaguagguuguaugg......................................................   435      0     seq
    ..........ugagguaguagguuguauggu.....................................................   8077     0     seq
    ..........ugagguaguagguuguaugguu....................................................   90093    0     seq
    ..........ugagguaguagguuguaugguuu...................................................   8916     0     seq
    ..........ugagguaguagguuguaugguuua..................................................   53       0     seq
    ..........ugagguaguagguuguauAguuua..................................................   131      1     seq
    ..........ugagguaguagguuguaugguuuagagu..............................................   1        0     seq
    ...........gagguaguagguuguau........................................................   4        0     seq
```

oar-let-7c

```
gcauccggguugagguaguagguuguaugguuuagaguuacacccugggaguuaacuguacaaccuucuagcuuuccuuggagc
...........gagguaguagguuguaug.......................................................   1    0    seq
...........gagguaguagguuguauggu.....................................................   1    0    seq
...........gagguaguagguuguaugguu....................................................   96   0    seq
...........gagguaguagguuguaugguuu...................................................   13   0    seq
............agguaguagguuguaugguu....................................................   1    0    seq
................................uagaguuacacccuggga..................................   1    0    seq
................................uagaguuacacccugggaguua..............................   3    0    seq
..........................................cccugggaguuaacuguacaaccuu.................   1    0    seq
..................................................guuaacuguacaaccuucuagcu...........   1    0    seq
.....................................................aacuguacaaccuucuagcuuu.........   2    0    seq
......................................................acuguacaaccuucuagcuuuc........   1    0    seq
.......................................................cuguacaaccuucuagcuuu.........   1    0    seq
.......................................................cuguacaaccuucuagcuuuc........   3    0    seq
.......................................................cuguacaaccuucuagcuuucc.......   23   0    seq
.......................................................cuguacaaccuucuagcuuuccu......   8    0    seq
........................................................uguacaaccuucuagcuuuccu......   2    0    seq
```
